# First Nations Australians and head and neck cancer: health professionals’ priorities for improving the pathway of care

**DOI:** 10.1007/s00520-025-09651-y

**Published:** 2025-06-25

**Authors:** Stephanie Ng, Elizabeth C Ward, Gail Garvey, Tamara Butler, Joanne Tesiram, Bena Brown, Jasmine Foley, Rebecca Packer, Aaron Hansen

**Affiliations:** 1 Centre for Functioning and Health Research, Metro South HHS, Brisbane, Australia; 2 School of Health and Rehabilitation Sciences, The University of Queensland, Brisbane, Australia; 3 School of Public Health, The University of Queensland, Brisbane, Australia; 4 Princess Alexandra Hospital, Metro South HHS, Brisbane, Australia; 5 Southern Queensland Centre of Excellence in Aboriginal and Torres Strait Islander Primary Healthcare, Metro South HHS, Brisbane, Australia; 6 Yardhura Walani, National Centre for Aboriginal and Torres Strait Islander Wellbeing Research, The Australian National University, Canberra, Australia; 7 Designates First Nations Research Collaborator, Brisbane, Australia

**Keywords:** First Nations Australians, Head and neck cancer, Concept mapping, Optimised pathway of care

## Abstract

**Purpose:**

I mprovements are needed in the care pathway for First Nations Australians with head and neck cancer (HNC); however, there is limited information to guide the development of culturally responsive care. The aim of this study was to use concept mapping to identify key priorities for service improvement in the HNC care pathway for First Nations Australians, through the perspectives of health professionals delivering care.

**Methods:**

Health care staff (*n* = 27, including four First Nations Australians) reflected on their care delivery experiences and generated suggested actions to improve HNC care for First Nations Australians. Participants then rated these statements for importance and changeability and grouped them into similar concepts. The data then underwent multivariate analysis and multidimensional scaling to identify major conceptual domains.

**Results:**

The final dataset included 73 unique statements, 21 from First Nations participants. Statements fell within nine cluster themes, in the following order of mean ranked importance: Person and family centred care, Continuity and care closer to home, Culturally safe care pathways, Staff cultural competency, Advocacy and support, Communication and connections, Culturally safe environment, Education and information, and Reducing financial burden. Of the 42 statements rated highest in importance, only 26 were perceived as both highly important and changeable, and eight of those were relating to improving Person and family centred care.

**Conclusions:**

Multiple areas for service improvement were identified, with varying levels of perceived changeability. The findings will inform further research involving co-design to enhance the care pathway for First Nations Australians with HNC.

**Supplementary Information:**

The online version contains supplementary material available at 10.1007/s00520-025-09651-y.

## Introduction

Aboriginal and/or Torres Strait Islander people, hereafter respectfully referred to as First Nations Australians, experience disproportionate burden and impact of head and neck cancer (HNC). First Nations Australians are almost twice as likely to be diagnosed with HNC [1], are significantly younger at diagnosis [2], and present with more advanced disease [3, 4]. In addition, they are much less likely to receive treatment, regardless of intent [5], and face significant delays from diagnosis to treatment [4]. First Nations Australians also experience worse survival than their non-First Nations counterparts [2], with 2.5 times the mortality rate in Queensland [6]. Whilst the reasons for these disparities have not been specifically explored for HNC, evidence around First Nations and cancer care more broadly highlights ongoing barriers to timely and appropriate healthcare access. These include challenges relating to travel and accommodation when having to leave Country for services [7, 8], communication barriers [7, 9], and ongoing discrimination and racism in the health system [10, 11]. Of note, factors that optimise cancer outcomes for First Nations Australians also require further attention and include provision of person-centred care that acknowledges self-determination and is inclusive of family and community, culturally safe care and communication, coordination of care, and holistic supportive care [11–13].

Achieving health equity for First Nations Australians has long been recognised as a priority and in recent years has had a renewed focus within the public health system in Australia [14], particularly within cancer care [15]. Despite health services and governing structures’ commitment to change, there is limited evidence about the care provided to First Nations people with HNC, making it difficult to implement practical and sustainable improvements. The aim of this study is to establish key priorities for service change based on the experiences of health professionals involved in caring for First Nations people with HNC.

## Methods

### Design

Concept mapping is a mixed methods approach that involves participants brainstorming and sorting ideas, which then undergo multidimensional scaling and cluster analysis to generate easily interpretable prioritised actions for change [16]. This participatory-based method engages diverse stakeholders who are directly impacted by the topic of the study [17] and is an approach that is well-utilised in the health setting for service design projects [16]. It has been shown to be effective across time periods and flexible with participants based across wide geographical distances [16, 18].

### Setting and context

The study is based in the state of Queensland, Australia. Queensland covers an area of 1,727,000 square kilometres and has a population of more than 5 million people [19]. As of 2021, over 237,000 First Nations people were living in Queensland [20], with 30% living within major cities, and the remaining majority dispersed widely across regional, rural, and remote locations [21]. All Queensland residents have access to healthcare through a free public health system delivered through 16 distinct Hospital and Health Services (HHS) responsible for providing care for those living in their respective geographical region. Metro South Health, where this research is conducted, covers the south side of the state capital city, Brisbane, and the surrounding Logan, Redlands, and Scenic Rim areas. The Princess Alexandra Hospital (PAH), which is part of Metro South Health, is recognised as one of the leading centres for HNC and skull base surgery in the state and is a tertiary referral centre for patients across Queensland, interstate, and from the Pacific Islands [22].

### Participants

Health professionals from public health services in Queensland providing care to First Nations patients with HNC and their caregivers were invited to participate in the study through snowball recruitment from the PAH Multidisciplinary HNC team. Target participants included those working in primary care, hospital care, and community/regional care pre-, during, and post-treatment, with priority given to staff who also identified as First Nations Australians. Twenty-seven participants were recruited, with four identifying as First Nations (15%). There was representation from a range of professions, predominantly from the metropolitan cancer service (67%), though most delivered care in more than one phase of the cancer continuum (74%) (Table 1).

**Table 1:** Participant demographics

Participant demographics	*n* (%)
Age	
18–35 years old	7 (26)
36–65 years old	19 (70)
65 + years old	1 (4)
Gender	
Male	8 (30)
Female	19 (70)
First Nations status	
Yes—Aboriginal	4 (15)
Yes—Torres Strait Islander	0 (0)
No—neither Aboriginal nor Torres Strait Islander	23 (85)
Profession	
Doctor	4 (15)
Nurse	7 (26)
Speech pathologist	6 (22)
Dietitian	4 (15)
Social worker	1 (4)
Radiation therapist	2 (7)
First Nations Liaison Officer	2 (7)
Community team leader	1 (4)
Years in profession	
< 10 years	7 (26)
10–15 years	7 (26)
> 15 years	13 (48)
Frequency of care delivery to First Nations patient with HNC	
Rarely or infrequent (e.g. 1–5 patients per year)	12 (44)
Often or very often (e.g. > 1–2 patients per month)	15 (56)
Location of care delivery	
Metropolitan hospital	18 (67)
Regional/rural hospital or health service	4 (15)
Primary care facility	3 (11)
Community outreach/home-based	0 (0)
More than one location	2 (7)
Care delivery phase (i.e. pre-treatment, treatment planning, treatment, post-treatment care/rehabilitation, surveillance, and palliation)	
Pre-treatment	5 (19)
Treatment planning	2 (7)
More than one phase of care	20 (74)

All participants completed Stages 1 and 2 of concept mapping, though four participants withdrew prior to Stage 3 as they were no longer available to participate, leaving 85% (23/27) who completed Stage 3. Whilst concept mapping is ideally completed with participants engaging in all three initial stages, this may not always be practical [17], and attrition between phases is often reported [23, 24].

### Concept mapping methods

Concept mapping is a process that involves six stages: (1) preparation, (2) statement generation, (3) structuring, (4) representation, (5) interpretation, and (6) utilisation [16]. Only the first five stages will be detailed in this study. Stage 6, which involves future utilisation of the current study data, together with results from a study exploring First Nations HNC survivors and their perceptions of current care, will inform a co-design study aiming to improve the care pathway for First Nations Australians.

### Data collection and analysis

#### Stage 1—preparation

Using semi-structured interviews, participants were encouraged to reflect on their experiences delivering care to First Nations people with HNC and their caregivers or families, across the cancer pathway. Discussion in the brainstorming session explored current practices, challenges, and opportunities for improving HNC care delivery to First Nations people, both within and outside their workplace. Staff were largely interviewed individually, with only two interviewing as a pair at their request. These sessions were done in person or virtually depending on their geographical location and preference. Interviews were conducted by the primary investigator (SN, a non-First Nations researcher and allied health clinician with experience in HNC care) and were recorded and transcribed verbatim for reference.

#### Stage 2—statement generation

The statement generation stage commenced immediately after the interviews within the same session. Based on ideas from the brainstorming, participants were guided to elicit a list of discrete statements they felt were specific and actionable steps needed to improve care delivered to First Nations people with HNC in their service. Once statements were collected from all participants, three research team members (SN, EW, BB) reviewed and consolidated them into a single list. Duplicate statements (those expressing the same idea) were combined into a single representative statement. When duplicate ideas were expressed in both First Nations and non-First Nations statements, the First Nations participant’s wording was retained to ensure their voices were preserved in the final dataset.

#### Stage 3—structuring

The final set of statements was returned to all participants individually, and participants were blinded to whom the statements originated from. They were asked to (a) sort these into conceptually similar groups and (b) rate each statement on two separate scales: importance: from 0 (not at all important) to 4 (extremely important) and changeability: from 0 (not at all changeable) to 4 (extremely changeable). The steps were done either in person or remotely.

#### Stage 4—representation and Stage 5—interpretation

Data from the structuring stage were entered into R-CMap, an open source and freely available concept mapping tool described by Bar and Mentch [25]. The input first underwent multidimensional scaling to generate a point map and stress value. A lower stress value reflects more reliable clustering and a goodness-of-fit measure of the diagram to the data [25, 26] with an upper acceptable limit of stress value reported to be 0.39 [27].

The next stage of analysis involved hierarchical cluster analysis to determine general concepts and themes based on how similar statements cluster together, represented as a dendrogram. Four members of the research team (SN, EW, BB, JF) reviewed the different cluster arrangements through an iterative process to ensure the final cluster solution best represented the grouping of similar statements into separate clusters that were conceptually distinct from each other. Two of the First Nations investigators (GG, TB) then reviewed the statements in each cluster and chose a descriptor that best represented each unique group of statements.

Cluster and statement data was then analysed and represented using three distinct visual representations generated from R-CMap: (1) a cluster rating map, showing (a) the proximity of statements, in which closer proximity reflects that statements were more frequently grouped together by the participants, (b) the size of the cluster, whereby smaller clusters indicate closer agreement in statements, and (c) the position of clusters relative to each other, with increased distance demonstrating that the cluster themes were more unique or unrelated to others; (2) an importance and changeability rating pattern-matching graph, which compares the average of all statement ratings regarding importance and changeability for each cluster and displays the relationship between these ratings; and (3) a Go-Zone plot, which is a bivariate plot with four quadrants in which individual statements are grouped depending on their relative overall importance and changeability rating. The green upper right quadrant, referred to as the ‘go-zone’, highlights the most important and most changeable statements as rated by participants.

## Results

A total of 282 statements were generated by all the participants. After the removal of duplicate statements, a final set of 73 unique statements remained, with 21 of those generated by First Nations staff (29%). The point map yielded a stress value of 0.339 which falls below the upper acceptable stress threshold, and so the model was deemed reliable.

**Fig. 1:**
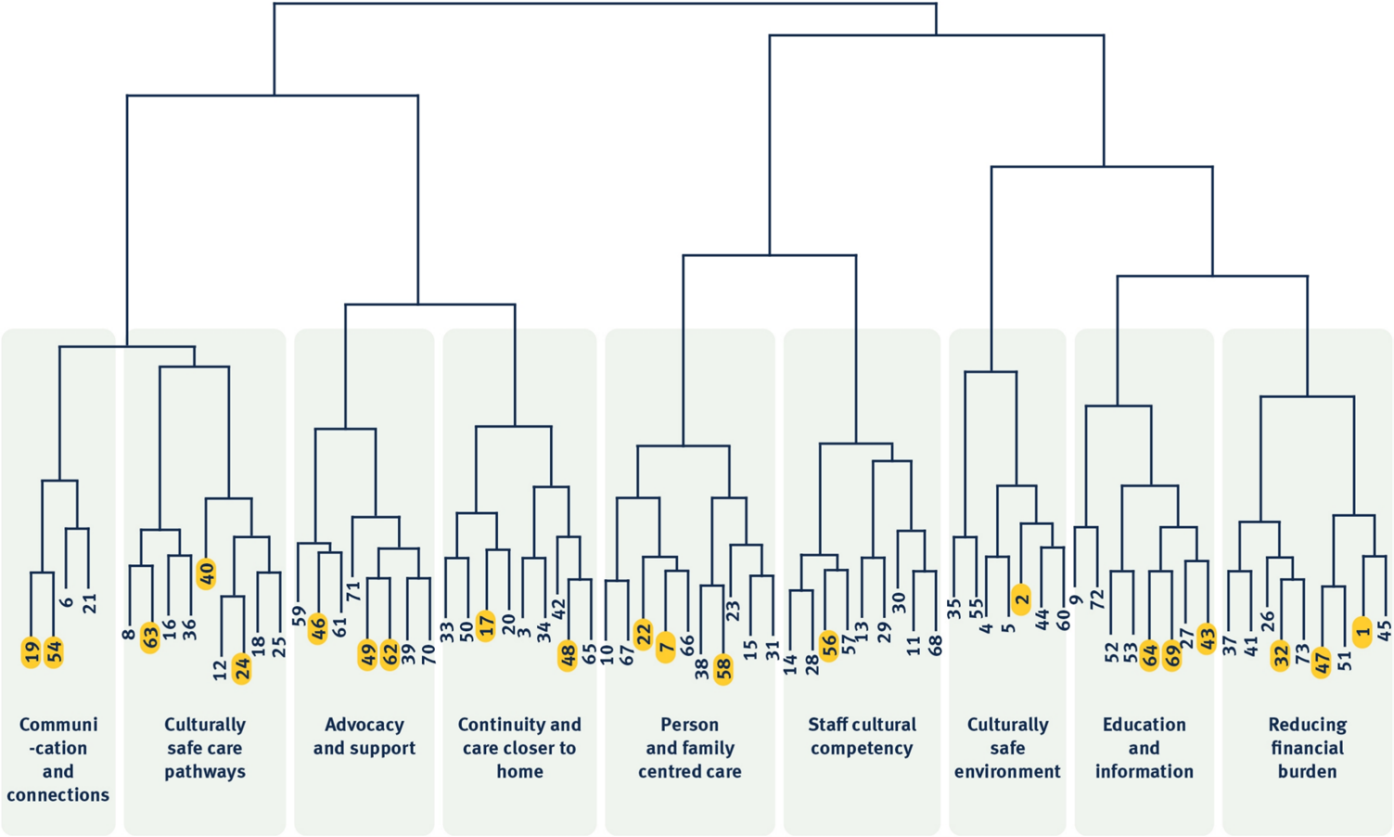
Dendrogram with the nine clusters. First Nations participants’ statements are highlighted in each cluster

Results of the hierarchical cluster analysis revealed nine unique clusters (Fig. 1). Descriptors of each cluster and exemplar statements are detailed in Supplementary File S1—Table 1. A notable observation was that First Nations participants’ statements, though fewer in number, organically spanned all identified clusters, suggesting broad alignment in identified priorities (Fig. 1).

**Fig. 2:**
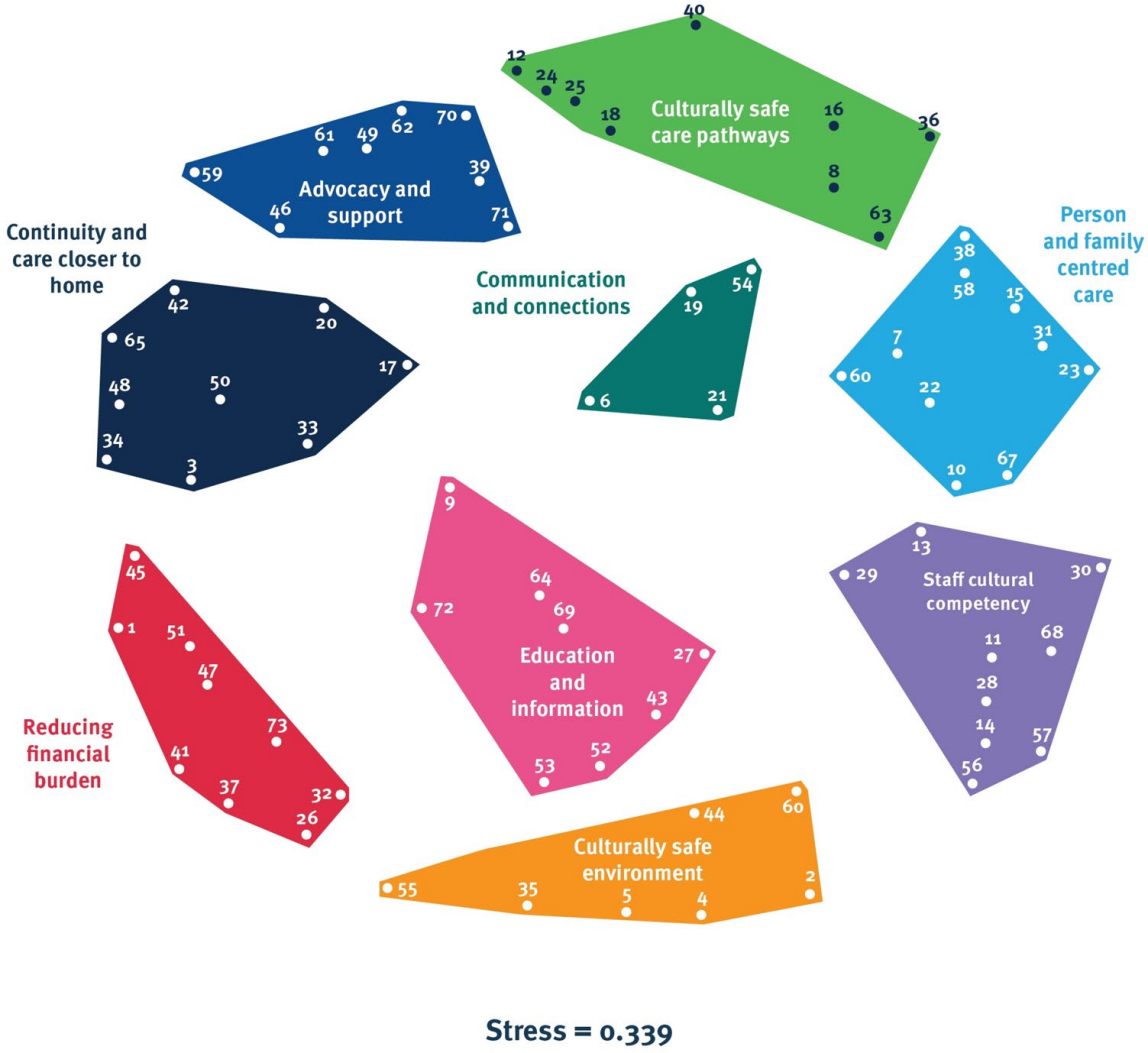
Cluster rating map

Next, the cluster rating map (Fig. 2) demonstrated that clusters were generally considered conceptually distinct as they were not abutting or overlapping on the visual representation. Most clusters were comparable in size; however, the Communication and connections cluster was smallest, indicating participants were highly consistent in grouping these statements together during the sorting task.

**Fig. 3:**
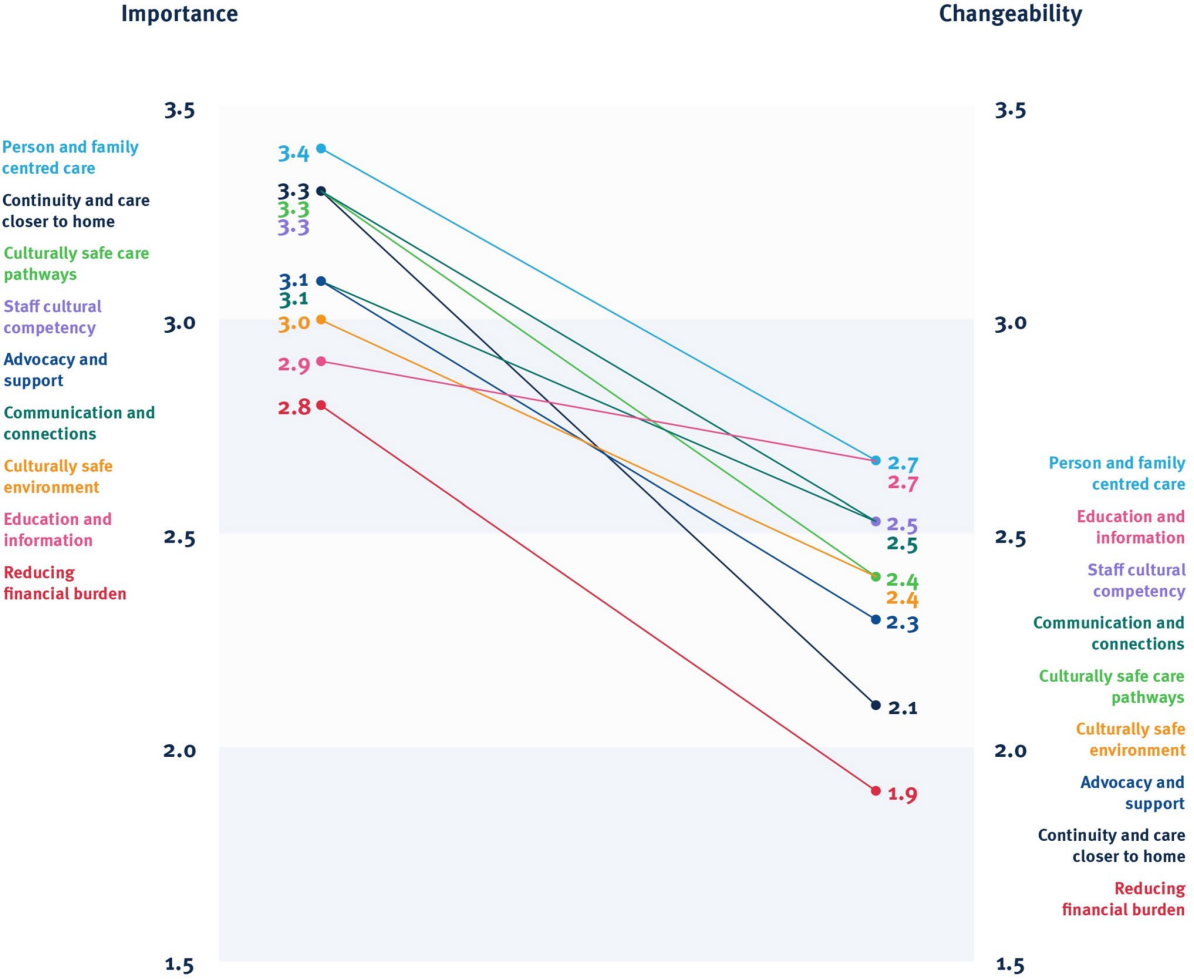
Importance and changeability rating pattern-matching graph

The pattern matching graph (Fig. 3) revealed Person and family centred care, Continuity and care closer to home, and Culturally safe care pathways were rated as the most important clusters, whilst Person and family centred care, Education and information, Staff cultural competency, and Communication and connections were rated the most changeable cluster themes. All clusters had a negative gradient, indicating participants perceived all clusters of suggested actions as being more important than changeable. Reducing financial burden was rated both the least important and least changeable cluster theme.

**Fig. 4:**
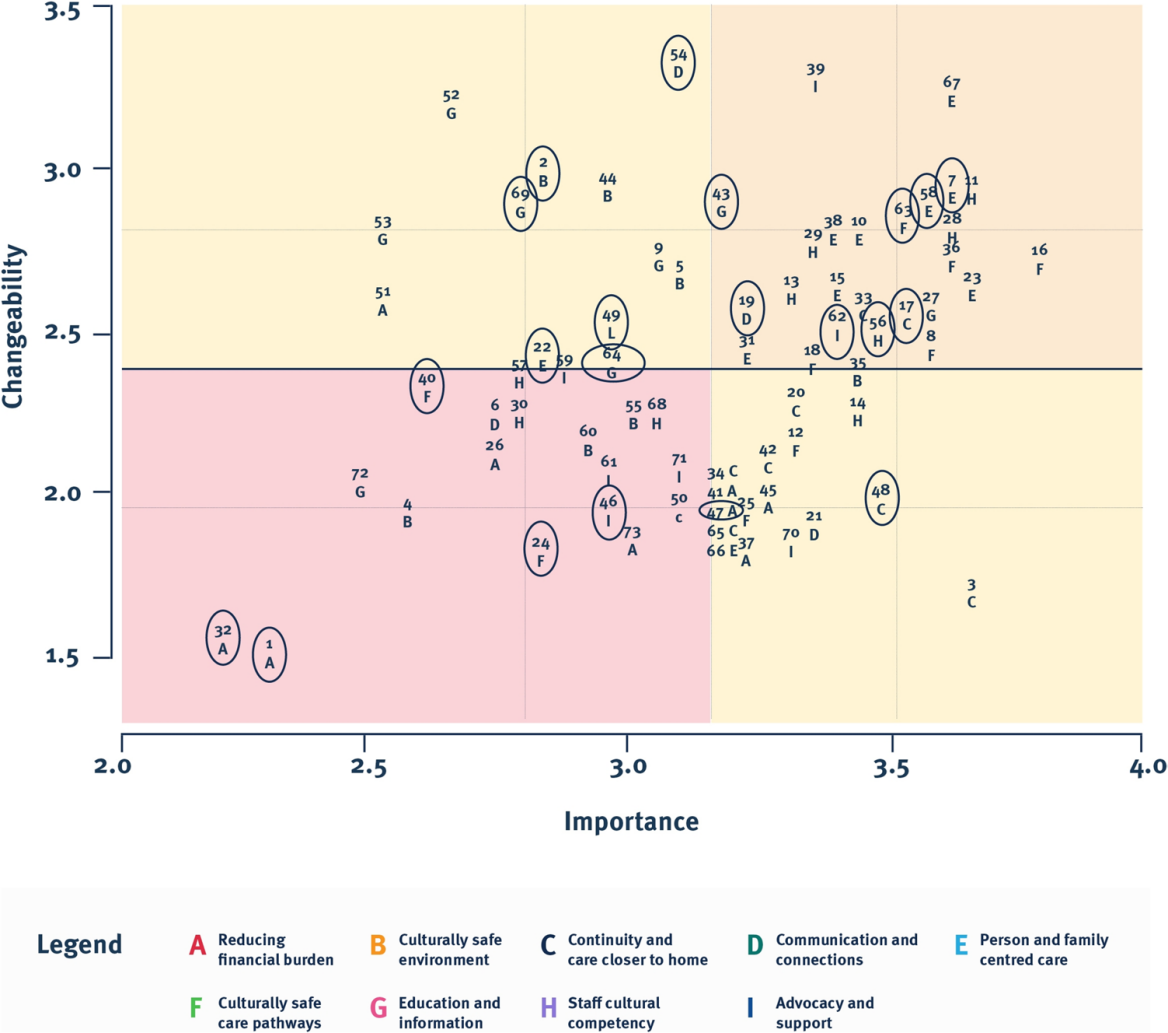
Go-Zone plot displaying statement numbers and corresponding cluster ID. First Nations staff statements are highlighted by circles

The Go-Zone plot revealed that 42 statements (58% of total), including 10 from First Nations staff, fell within the two quadrants on the right, representing those rated as highest in importance, regardless of changeability (Fig. 4). These statements originated from all clusters; however, Person and family centred care, Continuity and care closer to home, and Culturally safe care pathways were most represented. Statements rated as important but considered less changeable, i.e. within the right lower quadrant, were largely from Continuity and care closer to home cluster, e.g. statement 34: ‘Improve the skills of rural General Practitioners or rural Generalists to deliver care either at diagnosis phase (e.g. being able to do minor procedures like tonsil biopsies safely or nasendoscopy) as well as the surveillance phase’, followed by Reducing financial burden cluster, e.g. statement 41: ‘Mapping of any out of pocket costs and identify solutions, e.g. advocate to Medicare to increase the rebate for scans or offering the scans in public radiology’.

Specifically, within the go-zone, there was a total of 26 statements (36% of total), with eight of those being from First Nations staff participants (31% of go-zone statements) (Table 2). Go-Zone statements came from all clusters, except for the Reducing financial burden cluster. Patient and family centred care cluster was the most represented (*n* = 8, 31% of go-zone statements). The most important and changeable statement was number 67: ‘We need to accurately identify all First Nations patients on presentation’, followed by statement 11: ‘Current health professionals need to be aware of cultural and gender practices and respect cultural differences and preferences when delivering healthcare to First Nations people’.

**Table 2:** Go-Zone statements with mean importance and mean changeability ratings. First Nations participants’ statements are bolded

Cluster ID *	Cluster theme	Statement no	Statement	Mean importance	Mean changeability
B	Culturally safe environment	35	Improving public health and primary care programs around prevention and reducing risk factors and screening for head and neck cancer, e.g. alcohol, smoking, access to Gardasil/HPV vaccination	3.43	2.35
C	Continuity and care closer to home	**17**	**Ensuring that people not presenting for treatment are followed up by contacting community supports or GP**	**3.52**	**2.52**
		33	Improve telehealth access to reduce patient travel time and allow patients to stay with family and at home	3.43	2.57
D	Communication and connections	**19**	**Finding the best contact for the patient, e.g. support worker**	**3.22**	**2.52**
E	Person and family centred care	23	Giving patients autonomy and respect of allowing them to make their own health care decisions	3.65	2.61
		**7**	**Allow time for patients and family to digest information in the consult and give them time to ask questions**	**3.61**	**2.91**
		67	We need to accurately identify all First Nations patients on presentation	3.61	3.22
		**58**	**Staff to include the patient and their families and support networks in discussions and their care journey where possible**	**3.57**	**2.87**
		10	Culturally appropriate screening at head and neck clinic to identify wellbeing, needs, barriers and enablers to receiving care	3.43	2.78
		15	Engaging the patients to provide consumer feedback on current service and how to change current service to better support Indigenous population	3.39	2.61
		38	Involve family or most relevant or supportive family members or friends in patient’s care and ongoing support for family as well as for patients	3.39	2.78
		31	Identify the primary motivations for First Nations people receiving care and match care delivery to those priorities	3.22	2.43
F	Culturally safe care pathways	16	Ensure efforts are made for patients to get back to Country if that’s part of their wishes, particularly in the end of life	3.78	2.7
		36	In the palliative care phase for First Nations patients, we need to create a flexible environment where the patient has more of a say in the decision making of their care	3.61	2.7
		8	Clear and timely communication pathways (e.g. scans, surveillance advice, symptom management) between the primary and tertiary practitioners, and private and public systems, to ensure the patient gets what they need	3.57	2.48
		**63**	**To have clear communication about the appointments and encouraging First Nations patients to bring along a family member or kin family, or whoever they feel comfortable with**	**3.52**	**2.83**
		18	Every patient who identifies as First Nations automatically gets linked in with a First Nations liaison officer whose primary role is to connect with community and to be the patient’s guide from diagnosis to the very end of the journey	3.35	2.39
G	Education and information	27	Having culturally and linguistically safe information to provide to First Nations consumers, whether it’s written, video, graphic, e.g. for surgery, palliative care, food and fluids, treatment side effects, mouthcares, etc	3.57	2.52
		**43**	**More education around what clients should expect on their cancer journey e.g. pre-education before head and neck clinic, purpose of palliative care**	**3.17**	**2.87**
H	Staff cultural competency	11	Current health professionals need to be aware of cultural and gender practices and respect cultural differences and preferences when delivering healthcare to First Nations people	3.65	2.91
		28	Having relevant and applicable training for all staff to provide culturally safe care	3.61	2.83
		**56**	**Staff need to be aware of their own beliefs, ethos, biases, and show empathy**	**3.48**	**2.48**
		29	Health liaison officer providing regular inservices about their role, what they can do for patients, and how we can best utilise them to support the Indigenous population	3.35	2.74
		13	Education and access to interpreters to ensure patients understand medical information	3.3	2.61
I	Advocacy and support	**62**	**To have an Indigenous nurse navigator for head and neck cancer to follow through patient's journey with them via care coordination and linking in with support services, medical specialists, and family, internal or external providers**	**3.39**	**2.61**
		39	Liaison officers need training specifically to head and neck treatments and psychosocial impacts	3.35	3.26

*Cluster ID and themes alphabetised and ordered as per output from R−CMAP

The red-zone, which included statements rated lowest in importance and changeability, contained 17 statements (23% of total statements), with the cluster Reducing financial burden being the most represented (*n* = 4, 24% of red-zone statements). The statement considered least changeable and important was number 32: ‘If bringing patient down from rural remote, bring them down on pay day or the week of pay week’.

## Discussion

In this study, concept mapping enabled the identification of nine key areas where staff felt changes were needed to improve care delivery for First Nations people with HNC. These span across the HNC care continuum and involve changes to processes, cultural awareness, and the healthcare environment. Ratings of importance and changeability identified that staff felt a third of the suggested improvements could be prioritised as actionable changes.

Although over half of the statements were rated by staff participants as highly important to address, 26 (36%) fell within the go-zone, indicating actions that were considered both important but also most achievable to help make early, impactful change to care delivery. Most of these related broadly to staff cultural training and education, involvement of family in care delivery, more streamlined care coordination and follow up, improved education regarding the care journey, and respect for patients’ culture, autonomy, and wishes. These priorities align closely with health professionals’ service improvement recommendations from previous research within First Nations cancer care [7, 28, 29], as well as First Nations healthcare more generally [30]. The suggestions also mirror the principles outlined in the Optimal Care Pathway for Aboriginal and Torres Strait Islander people with cancer [12], which are underpinned by evidence-based guidelines and concepts in the National Aboriginal and Torres Strait Islander Cancer Framework [31], and supported by the Aboriginal and Torres Strait Islander Cancer Plan [32].

Person and family centred care cluster was rated as the most important and one of the most changeable clusters. These staff priorities are consistent with the literature in First Nations cancer care, whereby family and caregivers are frequently identified as facilitators and a source of strength for those accessing care, particularly in their roles as the patient advocate, mediator between the patient and broader family, and the provider of emotional support and practical assistance [8, 11, 13]. Many of these statements also closely reflect the principle of Patient-Centred Care in the Optimal Care Pathway, which highlight the need to acknowledge First Nations philosophies of holistic health, including social and emotional wellbeing, and emphasise family engagement in maintaining connections with the patient and shared informed decision making [12]. Recently developed culturally appropriate tools to measure wellbeing for First Nations people, such as Good Spirit, Good Life [33], and What Matters 2 Adults [34], may warrant more routine utilisation in the future to support individualised and tailored HNC service delivery. This is particularly in line with statement 10: ‘Culturally appropriate screening at head and neck clinic to identify wellbeing, needs, barriers and enablers to receiving care’. Of note, the statement rated most important and changeable ‘We need to accurately identify all First Nations patients on presentation’ is also a key target within the aforementioned national guidelines. It is known that misidentification of First Nations patients in Queensland public hospitals occurs approximately 12% of the time [35] and ensuring appropriate identification reflects the first step in delivering culturally competent care.

Of the statements rated as important but less changeable, most were from the Continuity and care closer to home cluster. The key available health services being spread across wide geographical distances relative to the patients in Queensland can create unique challenges and complexities with health care provision, particularly in cancer care. Current evidence around HNC management, as well as within First Nations cancer care, highlights that patients living in regional and rural areas have limited access to local primary health and specialist services [10], fewer multidisciplinary team reviews [3], increased likelihood of having to travel away from local health services for treatment [3], and patients have lower survival with increasing rurality [3]. Given the proportion of First Nations people increases in more rural and remote regions [36], they may be disproportionately affected by such geographical challenges. Furthermore, cultural factors, such as kinship responsibilities, traditional roles, and connection to Country, may require additional consideration for First Nations people who are already grappling with a HNC diagnosis and need for treatment [10]. Given the long-term wider system level changes and significant funding implications that may be required to address this health access barrier, the proposed related actions are likely viewed as less easily and quickly implementable within an optimised care pathway. However, some recommendations by participants include improving access to telehealth to reduce patient travel and connecting with community services and GP to support local care, which may represent small steps towards change.

Unsurprisingly, all clusters were rated as more important than changeable. Healthcare is a complex and dynamic system, and translation of research into clinical practice is known to be challenging [37]. Previous research on health professionals’ perspectives on quality improvement highlighted a number of barriers, including time constraints, resource limitations, competing demands, reduced management support, or perceived lack of authority or skill to create change [38]. This is likely consistent with the Reducing financial burden cluster being seen as the least actionable and lowest priority cluster amongst the rest. Logistical and practical support to implement change, as well as tailored professional behaviour change strategies for health professionals, such as those outlined by Grimshaw et al. [37], should be considered within the utilisation stage.

## Limitations

A lack of representation from Torres Strait Islander health professionals was a limitation in this study, and greater participation by First Nations staff would have strengthened the cultural validity of the findings. First Nations health professionals and students experience, amongst other existing systemic barriers, high levels of racism in the health system, and this contributes to ongoing exceptionally low numbers entering the health care sector [39]. Although First Nations staff comprised 15% of the cohort, this is higher than the known representation of First Nations people within the Queensland Health workforce (1.67%) [40] and in the general population (3.8%) [41]. Given the small numbers, efforts were made in the analysis process to ensure First Nations staff statements were privileged, and mapping of statements across the clusters revealed priorities identified by both First Nations and non-First Nations staff were broadly aligned.

Concept mapping is known to be a ‘best-fit’ representation of recommendations and naturally may not always be a perfect fit [42] but rather reflects a strength in its ability to capture diverse stakeholder perspectives. Previous use of concept mapping within First Nations health research, both locally in Australia [42, 43] as well as overseas [44], demonstrated its value in promoting community engagement and ownership of the research, as well as being highly adaptable to First Nations contexts [45]. Importantly, this is the first known use of this methodology within First Nations cancer research, and specifically within HNC. It should be noted that First Nations staff may have felt hesitation to openly reflect on their perspectives and experiences, particularly to a non-First Nations facilitator. Subsequent studies within the larger project will ensure First Nations patient, carer, and staff voices are thoroughly represented through other culturally accepted methodologies, i.e. Yarning and co-design, and involve a First Nations facilitator. All perspectives will then inform the co-design of HNC pathway improvements for First Nations Australians; however, as this research relates to public health services of a single Australian state, the generalisability and applicability of findings for other states and services require verification.

## Conclusion

Concept mapping facilitated the conceptualisation and prioritisation of actions that staff perceive are needed for service improvement. It enabled understanding of recommendations from diverse staff stakeholders, from various clinical backgrounds and health services. Broad priorities for change were identified, and the go-zone with the 26 suggested actions rated as highest importance and changeability will be shared in the future co-design process with First Nations stakeholders to help inform positive changes to the care pathway for First Nations people with HNC.

## Supplementary Information

Below is the link to the electronic supplementary material.

ESM 1 DOCX (23.2 KB)

## Data Availability

No datasets were generated or analysed during the current study.
