# Supplementary material for: First Nations Australians and head and neck cancer: health professionals’ priorities for improving the pathway of care
Source: Support Care Cancer. 2025 Jun 25;33(7):624. doi: 10.1007/s00520-025-09651-y (PMC12187825; doi:10.1007/s00520-025-09651-y)
Supplement: Supplementary file 1 — DOCX (23.2 KB) [file 520_2025_9651_MOESM1_ESM.docx]

**Supplementary File S1**

**Table 1** Cluster themes and descriptions with mean importance and changeability. Exemplar statements for each cluster theme are included with First Nations staff statements bolded.

| Cluster ID * | Cluster themes | Cluster theme description | Exemplar statement | No. of statements | Mean Importance | Mean Changeability |
| --- | --- | --- | --- | --- | --- | --- |
| A | Reducing financial burden | Finding solutions for costs and supports | **More options and staffing for transport to hospital from greater Brisbane and locally, e.g. cab vouchers, courtesy or shuttle bus from the airport (statement 47)** | 9 | 2.845 | 1.908 |
| B | Culturally safe environment | Creating a culturally safe space and care delivery | Access to information about culturally appropriate foods e.g. high protein foods, how to thicken up culturally appropriate drinks (statement 5) | 7 | 2.969 | 2.447 |
| C | Continuity and care closer to home | Improving and increasing local or virtual care | **More outpatients, hospital in the home, or in-home services to support head and neck cancer patients post treatment (statement 48)** | 9 | 3.343 | 2.106 |
| D | Communication and connections | Improving flexibility and options for contact | Flexibility, patient-focussed outpatient bookings, i.e. timely appointment letters and repeated efforts at contact rather than cut people off because they've not responded twice (statement 21) | 4 | 3.098 | 2.478 |
| E | Person and family centred care | Personalised care that involves family or support networks | **For staff to put more information of the patient's story or journey in the handover to the community, not just the diagnosis but how the patient presented or if any extra support needed (statement 22)** | 10 | 3.387 | 2.657 |
| F | Culturally safe care pathways | Timely and co-ordinated follow up care | **Linking patients who are well enough to community groups to keep them active in the community (statement 40)** | 9 | 3.309 | 2.372 |
| G | Education and information | Creating a range of appropriate education resources | Create a document, e.g. lists and maps, with all the community support services specific to the Indigenous population (that are available that might be able to support feeds, thickened fluids, and therapy), and can be Queensland wide (statement 9) | 8 | 2.897 | 2.668 |
| H | Staff cultural competency | Embedding cultural training in the workforce | We need to acknowledge bush medicine, cultural treatments and healing play an important part for a lot of our patients (statement 68) | 9 | 3.271 | 2.517 |
| I | Advocacy and support | Presence of First Nations staff in supportive roles | We need to increase the number of liaison officers to make them accessible and be present for all patients at every point of their health journey (statement 70) | 8 | 3.109 | 2.315 |

^* Cluster ID and themes aphabetised and ordered as per output from R-CMAP.^
